# Visual capture and the experience of having two bodies – Evidence from two different virtual reality techniques

**DOI:** 10.3389/fpsyg.2013.00946

**Published:** 2013-12-18

**Authors:** Lukas Heydrich, Trevor J. Dodds, Jane E. Aspell, Bruno Herbelin, Heinrich H. Bülthoff, Betty J. Mohler, Olaf Blanke

**Affiliations:** ^1^Center for Neuroprosthetics, School of Life Sciences, Ecole Polytechnique Fédérale de LausanneLausanne, Switzerland; ^2^Laboratory of Cognitive Neuroscience, Brain Mind Institute, School of Life Sciences, Ecole Polytechnique Fédérale de LausanneLausanne, Switzerland; ^3^Department of Neurology, University HospitalGeneva, Switzerland; ^4^Human Perception, Cognition and Action, Max Planck Institute for Biological CyberneticsTübingen, Germany; ^5^Department of Brain and Cognitive Engineering, Korea UniversitySeoul, South Korea

**Keywords:** heautoscopy, full body illusion, virtual reality, ownership, self consciousness

## Abstract

In neurology and psychiatry the detailed study of illusory own body perceptions has suggested close links between bodily processing and self-consciousness. One such illusory own body perception is heautoscopy where patients have the sensation of being reduplicated and to exist at two or even more locations. In previous experiments, using a video head-mounted display, self-location and self-identification were manipulated by applying conflicting visuo-tactile information. Yet the experienced singularity of the self was not affected, i.e., participants did not experience having multiple bodies or selves. In two experiments presented in this paper, we investigated self-location and self-identification while participants saw two virtual bodies (video-generated in study 1 and 3D computer generated in study 2) that were stroked either synchronously or asynchronously with their own body. In both experiments, we report that self-identification with two virtual bodies was stronger during synchronous stroking. Furthermore, in the video generated setup with synchronous stroking participants reported a greater feeling of having multiple bodies than in the control conditions. In study 1, but not in study 2, we report that self-location – measured by anterior posterior drift – was significantly shifted towards the two bodies in the synchronous condition only. Self-identification with two bodies, the sensation of having multiple bodies, and the changes in self-location show that the experienced singularity of the self can be studied experimentally. We discuss our data with respect to ownership for supernumerary hands and heautoscopy. We finally compare the effects of the video and 3D computer generated head-mounted display technology and discuss the possible benefits of using either technology to induce changes in illusory self-identification with a virtual body.

## INTRODUCTION

In neurology and psychiatry the detailed study of illusory own body perceptions (Lhermitte, 1939; Hécaen and Ajuriaguerra, 1952; Critchley, 1953; Ionasescu, 1960) has suggested close links between bodily processing and self-consciousness. Such illusions include a variety of alterations in perceptual bodily experience such as the experiences of the absence of a body part (Hécaen and Ajuriaguerra, 1952; Critchley, 1953; Frederiks, 1969), body part transformations (Lhermitte, 1939; Hécaen and Ajuriaguerra, 1952; Lippman, 1952; Ionasescu, 1960), body part displacement (Lippman, 1952; Nightingale, 1982), disconnection of one body part from the body (Lippman, 1952; Blanke et al., 2003; Heydrich et al., 2010), the delusional misidentification of one’s own body part (i.e., somatoparaphrenia; Gerstmann, 1942; Vallar and Ronchi, 2009), as well as phantom limbs (Lhermitte, 1939; Hécaen and Ajuriaguerra, 1952; Brugger, 2006), and supernumerary phantom limbs (Vuilleumier et al., 1997; Khateb et al., 2009; for reviews, see Blanke et al., 2008; Blanke, 2012). Dramatic forms of illusory own body perceptions are autoscopic phenomena, such as the out-of-body experience and the doppelganger experience (also known as heautoscopy) of neurological origin (Brugger, 2002; Blanke et al., 2004). During autoscopic phenomena patients not only report altered perceptual bodily experience, but a breakdown of the spatial unity between the physical body and the self (Brugger, 2002). Thus, patients suffering from an out-of-body experience perceive the self and the world as if their self was localized outside their physical bodily boundaries (abnormal self-location and first person perspective) and no longer identify themselves with their physical body but with a spatial position where the illusory autoscopic body is perceived (abnormal self-identification; Blanke and Metzinger, 2009). While out-of-body experiences are disturbing experiences, patients continue to experience the self as singular, that is, they experience only one self that is abnormally localized, but at one single position and vantage point (Brugger, 2002). This is not the case in many patients suffering from heautoscopy, where the self may be experienced as reduplicated – existing at two or even more locations simultaneously (for review, see Brugger et al., 2006). Thus, in heautoscopy the self may be experienced at the location of the physical body, at that of the autoscopic body, or self-location may be at both locations at the same time (Brugger, 2002; Blanke et al., 2004; Heydrich and Blanke, 2013).

How can we study the mechanisms of experienced singularity of self-consciousness in healthy participants? Virtual and augmented reality techniques have enabled scientists in recent years to study many aspects of human behavior and multi-sensory integration. Scientists have used virtual reality (VR) techniques to investigate visual capture of the arm or the entire body (Slater et al., 2009; Sanchez-Vives et al., 2010; Slater et al., 2010), and for a broad range of research topics such as phobias [fear of public speaking (Slater et al., 2006), heights (Usoh et al., 2003), tunnels (Mühlberger et al., 2007), social interaction (Bailenson et al., 2003), proxemics (Llobera et al., 2010), visual control of locomotion (Warren et al., 2001; Mohler et al., 2007), and space perception (Thompson et al., 2004; Mohler et al., 2010)]. Use of VR techniques has also enabled scientists to carefully control stimuli that are otherwise difficult or even impossible to control in the real world.

Virtual reality can be used in combination with video technology (often referred to as augmented reality) or can be used with a 3D graphical rendering of a surrounding visual world. Different research groups have recently developed experimental paradigms to study several aspects of bodily self-consciousness – such as full body ownership or self-identification with the hand or body, as well as self-location (i.e., the experience of where I am in space) – by exposing participants to multisensory conflicts of bodily stimuli (Botvinick and Cohen, 1998; Ehrsson, 2007; Lenggenhager et al., 2007; Ionta et al., 2011; Blanke, 2012; Aspell et al., 2013). Initiated by seminal work on illusory ownership for a fake hand (e.g., rubber hand illusion, Botvinick and Cohen, 1998; Schmalzl and Ehrsson, 2011) more recent work has targeted illusory ownership for a virtual body (e.g., the full body illusion, see below, Ehrsson, 2007; Lenggenhager et al., 2007), a virtual face (Tsakiris, 2008; Sforza et al., 2010) or a virtual voice (Zheng et al., 2011).

Thus, it has been found that during the full body illusion participants self-identify with a virtual body and show drifts in self-location towards the virtual body if their back is stroked while they see their own virtual body on a head mounted display (HMD) being stroked in synchrony (Lenggenhager et al., 2007, 2009; Ionta et al., 2011). These findings were strengthened by Aspell et al. (2009), who showed that this experimental scenario is associated with changes in visuo-tactile perception as quantified by the crossmodal congruency effect (CCE). Despite the importance of these studies for the understanding of self-identification and self-location, these experimental setups did not alter the experienced singularity of the self, as they were mostly inspired by studies of out-of-body experiences (Ehrsson, 2007; Lenggenhager et al., 2007). However, the related neurological condition of heautoscopy is associated not just with altered self-identification and self-location, but frequently with a loss of experienced singularity of the self leading to self-identification with two spatially distinct bodies and self-location at two distinct spatial locations (bi-location of the self; Lenggenhager et al., 2007, 2009; Aspell et al., 2009). As this latter important aspect of bodily self-consciousness has not been studied in earlier experimental work, we here modified the full-body illusion based on a recent experiment that induced illusory ownership for two fake hands that are being stroked synchronously with the hidden hand of the participant (Ehrsson, 2009; Guterstam et al., 2011). While these rubber hand studies were able to demonstrate that supernumerary extremities can be experienced as “mine,” as in the clinical condition of supernumerary phantom limbs (13, 14), we here investigated whether we could experimentally induce self-identification with more than one body.

Running two studies using two different techniques, e.g., video (study 1) and computer generated graphics (study 2), also allowed us to compare the two approaches. On the one hand, 3D computer generated graphics enables the experimenter to control the representation of participants. The experimenter can manipulate, for example, body size (Yee and Bailenson, 2007), identity (Yee et al., 2009), or the motion characteristics of the body (Ries et al., 2009). Video, on the other hand, preserves the visual cues from the real world, including the identity of the participants.

Moreover, in experiments investigating multi-sensory stimuli, precise timing is likely important. Typically in video the delay between the seen image in the HMD and what one would see without wearing the HMD is low and well controlled, whereas in 3D computer generated graphics, this time additionally depends on the tracked data and is typically between 25 and 60 ms. Thus, it might be useful for the field of VR to compare 3D computer generated and video display of humanoid avatars according to new criteria, oriented towards the subjective feeling of self-identification with and self-location towards a virtual body rather than the usual criteria of visual realism.

To summarize, the present studies aimed to investigate whether healthy participants may experience a breakdown of the experienced singularity between the body and the self as quantified through self-identification with more than one body in our different VR setups. This allowed us to compare the two VR techniques that are most widespread today (video-based VR and computer generated-based VR) using the same paradigm. We first extended a previous setup (Lenggenhager et al., 2007) using a video HMD (study 1) and investigated self-location and self-identification while participants saw two fake bodies that were stroked either synchronously or asynchronously with their own body. We hypothesized that self-identification with two fake bodies would be stronger during synchronous stroking and that self-location would be significantly shifted towards the two bodies in the body synchronous (BS) condition only. Furthermore, we hypothesized that synchronous stroking will be associated with the breakdown of the singular self and the feeling of having multiple bodies. In a second follow-up study we adapted the set-up to 3D computer generated graphics in order to compare video with computer generated technology (Study 2) using a between subject design maintaining the same hypotheses as in study 1.

## MATERIALS AND METHODS

### PARTICIPANTS

A total of 19 healthy right-handed participants took part in study 1 (11 males, mean age 24.7 years) and 17 healthy participants took part in study 2 (9 males, mean age 27.8 years). All participants had no previous experience with the task or related experimental paradigms. All participants had normal or corrected to normal vision and had no history of neurological or psychiatric conditions.

### ETHICS STATEMENT

All participants gave written informed consent and were compensated for their participation. The study protocol was approved by the local ethics research committee – La Commission d’éthique de la recherche Clinique de la Faculté de Biologie et de Médécine – at the University of Lausanne, Switzerland (study 1) and the ethical committee of the University of Tübingen (study 2), and was performed in accordance with the ethical standards laid down in the Declaration of Helsinki.

### STUDY 1

#### Materials

**FIGURE 1:**
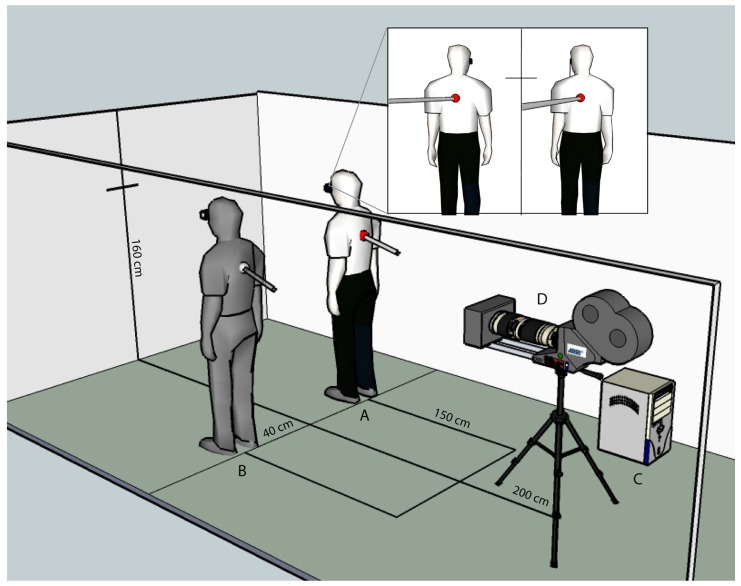
**Setup Study 1.** Participants stood at a distance of 2 m and with their backs facing a video camera. Participants were either standing to the right or the left of the midline. **(A)** Refers to the location of the participant, **(B)** to the perceived location of the duplicated image of the participant, **(C)** to the splitter that duplicates the video image, and **(D)** to the video camera which is used to project the video image into the head-mounted display.

Participants stood at a distance of 2 m and with their backs facing a video camera (JVC Victor GR-X5 3CCD, 30 degree field of view). Participants were either standing to the right or the left of the camera (see **Figure 1**). The video image was cropped at the midline and subsequently duplicated using a Sony DFS-300, DME switcher (Sony Corp., Tokyo, Japan). The video was projected in real time (<16 ms, except for asynchronous blocks, see below) onto a HMD (Virtual Viewer 3D, SVGA 80 × 600, 35 degree field of view, Virtual Realities Inc.) enabling participants to view their body once on the left and once on the right in a split screen mode (body condition). White noise was presented over headphones to mask any noise, and participants wore a cloth hood over their heads to occlude vision of their surroundings. The experiment took place under constant artificial illumination. During “stroking blocks” the backs (the area spanning the shoulders to waist) of participants were irregularly stroked, about once every 2 s by the experimenter with a long wooden stick, and participants viewed their body and the stroking via the HMD. The blocks lasted about 2 min. In asynchronous blocks a camera delay of 400 ms was introduced (DelayLine, Ovation Systems Ltd., London, UK). As a control condition a white upright rectangular human-sized metal panel (object condition) was chosen. All stimulus and procedural details were as described for the synchronous and asynchronous blocks in the body condition, except that in “synchronous object” blocks, participants’ backs were stroked with the stick in synchrony with stroking viewed – via the HMD – on the object. In the “asynchronous object” blocks the participants’ backs were again stroked with the stick but a delay was added to the visual display presented on the HMD so that the “felt stroking” was asynchronous with respect to the seen stroking on the object.

#### Procedure

The procedure was identical for all blocks except for those details added below. Participants were instructed to keep their eyes open and fixate the cross in between the two bodies, as viewed via the HMD (see **Figure 1**). In order to accurately assess self-location during the four conditions, the procedure of the drift measurement was practiced while wearing the HMD but before starting the experiment. This was done to make sure that each participant was comfortable with blindfolded walking. At the end of the block (of duration 2 min) self-location was measured once by first passively displacing the participants (the experimenter gently guided the participants while they took very small steps backwards for approximately 1.5 m). They were then asked to walk back to their initial position (while still blindfolded) with normal-sized steps (as in Lenggenhager et al., 2007). The distance (the “drift”) between the position held during the experimental block and the position estimated by the participant was measured in the anterior–posterior and left–right axis by hand with a measurement tape. Self-identification with the seen body and other phenomenological aspects were assessed at the end of each block by a written questionnaire adapted from Lenggenhager et al., (2007; see **Table 1**), where subjects had to rate each item on a seven-point Likert scale (1 = “I don’t agree at all”; 7 = “I totally agree”). Additionally, after each block participants were asked if they identified more with the right or the left body/object, or both equally. Participants took a short break before the subsequent block if requested. The order of blocks as well as the initial position (either to the right or the left of the midline) were randomized and counterbalanced across participants.

**Table 1:** Questions for Studies 1 and 2.

**Questions for Study 1 and Study 2**
(1) It seemed as if I were feeling the touch of the stick in the location where I saw the two virtual bodies/objects touched. (2) It seemed as though the touch I felt was caused by the stick touching the two virtual bodies/objects. (3) I felt as if the two virtual bodies/objects were my body. (4) It felt as if my (real) body was drifting forwards (towards the two virtual bodies/objects). (5) It seemed as if I might have more than one body. (6) It seemed as if the touch I was feeling came from somewhere between my own body and the two virtual bodies/objects. (7) It appeared (visually) as if the two virtual bodies /objects were drifting backwards (towards my body). (8) Did you identify with … (1) The right virtual body/object more; (2) The left virtual body/object more; (3) Both virtual bodies/objects equally
**Additional Question for Study 2 only: if answered 3 to question 8**
(9) Did you identify with the two avatars/objects … (1) At the same time; (2) At different times
*(Virtual bodies (study 1)/avatars (study 2) or objects was used in all questions depending on condition)

### STUDY 2

#### Materials

**FIGURE 2:**
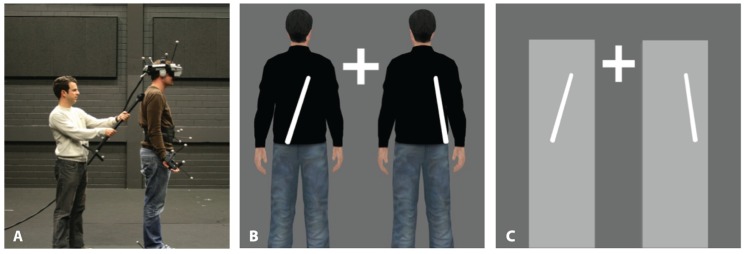
**Setup and visual stimulus Study 2.** **(A)**. Experimenter stroking a participant who is wearing an HMD, and tracking objects for feet, hands, and torso. The subject of the photograph has given written informed consent to publication of their photograph. **(B)** Visual stimulus projected in stereo in the HMD for the body condition. **(C)** Visual stimulus projected in stereo in the HMD for the object condition.

Participants stood, always at the same location, in a large tracking hall wearing markers on their hands, feet, head, and pelvis to track their positions using 16 Vicon MX13 cameras. They wore a HMD (nVisor SX60, 1280 × 1024 per eye, stereo, with 44 × 35 degree field of view) and viewed a stereoscopic computer generated image of either two identical avatars (body condition) or two identical objects (object condition) positioned 3 m in front of them. The avatars or objects were scaled to match the width and height of the participants. Graphics were presented in real-time with a latency of 40 ms. Participants were instructed to remain still when standing, but if they moved the virtual characters’ limbs would also move in accordance with the tracked limbs of the participants. In the case of the object condition, if the participants moved slightly the object location was also updated based on torso movement. In the asynchronous condition, the movements of the avatars/objects were also delayed. This was so that it corresponded exactly to the previous experiment (study 1) using video HMD, and minor movements of participants (e.g., swaying) were represented. White noise was presented over headphones to mask any noise, and participant’s vision of their surroundings was occluded by the HMD setup (black felt within the nVisor HMD). The 3D-computer generated environment had a constant artificial illumination (see **Figure 2**). During “stroking blocks” the back (the area spanning the shoulders to waist) of participants were irregularly stroked, about twice per second by the experimenter with a long stick, and participants viewed the stroking as rendered via the HMD. The blocks lasted about 2 min. In asynchronous blocks a delay of 400 ms of the virtual stick’s tracking information was introduced so that “seen stroking” and “felt stroking” did not correspond. For the control condition we used a white upright rectangular human-sized virtual object (object condition, see **Figure 2**). All stimulus and procedural details were as described for the synchronous and asynchronous blocks in the body condition, except that in “synchronous object” blocks, participants’ backs were stroked with the stick in synchrony with stroking viewed – via the HMD – on the object. In the “asynchronous object” blocks the participants’ back was again stroked with the stick but a delay was added to the visual display presented on the HMD so that the “felt stroking” was asynchronous with respect to the seen stroking on the object.

#### Procedure

The procedure was identical to study 1, with the exception that in this experiment the participants saw either two computer generated characters (matched gender) or two computer generated blocks. The procedure was identical for all blocks except for those details added below. Participants were instructed to keep their eyes open and fixate the cross in between the two bodies, as viewed via the HMD (see **Figure 2**).

For the drift measure, a training phase allowed participants to practice the blind walking before the experiment began. They practiced first without the HMD, then with the HMD using a top-down camera to show them where they were in relation to their original starting point. During the study, participants were stroked for 2 min, then their displays went blank, and self-location was measured as in study 1. All the motion tracking data were recorded to disk, so the trajectories and distances walked by participants were available for analysis. The drift was measured in the anterior–posterior and the left–right axis. As in study 1, the participants were not given feedback about their performance and instead were blindly moved around a 3 m × 3 m area to be returned to their physical starting location for the next trial.

The questionnaires (see **Table 1**) appeared one at a time on the HMD displays, and participants were given a Nintendo Wiimote to select their answers on a continuous scale between 1 ( = “I don’t agree at all”) and 7 ( = “I totally agree”) with scores accurate to two decimal places. Thus, they did not need to remove the HMD between conditions (unlike Study 1 where a written questionnaire was used). For study 2, an additional question concerning the temporal aspects of the identification with the avatar/objects was asked. If they answered that they identified with both avatars equally (Question 8: “Did you identify with the right, the left or both virtual bodies/object equally”), they were asked an additional question: did you identify with both avatars at the same time, or at different times? (see question 9, **Table 1**). Participants took a short break before the subsequent block if requested. The order of blocks were randomized and counterbalanced across participants.

### STATISTICAL ANALYSIS

Statistical analyses within each study were conducted as follows. The drift (self-location) measure (anterior–posterior drift and left–right drift calculated relative to initial position) was analyzed using multilevel linear mixed effects models with within-subjects factors *body* (body/object) and *synchrony of stroking* (synchronous/asynchronous) as random effects nested within subjects (Field et al., 2012). The questionnaire scores were (a) not normally distributed (Shapiro–Wilk test on model residuals) and (b) of ordinal structure, and so we analyzed the questionnaire data using the Wilcoxon signed-rank tests with planned comparisons between the following conditions: BS vs. body asynchronous (BA), object synchronous (OS) vs. object asynchronous (OA), and BS vs. OS, and BA vs. OA. The significance level used was 0.05, corrected for multiple comparisons using the Holm–Bonferroni method.

The responses to question 8 (“Did you identify with the right body more, the left body more or with both bodies equally?”) were considered spatially, with *left body more *< *both bodies equally *< *right body more*, hence the responses were on an ordinal scale. We tested for an effect of *body* and *synchrony* on participants’ responses using proportional-odds logistic regression (McCullagh and Nelder, 1989; Venables and Ripley, 2002).

An in-depth between-groups statistical analysis proceeds the results of each study. This provides a statistical comparison between the video-based (study 1) and 3D computer generated (study 2) VR techniques.

## RESULTS

### STUDY 1

We found that self-identification with the two virtual bodies and objects depended on synchrony. This was associated with the sensation of having more than one body, which was strongest in the body synchronous condition. Self-location, measured by the mean drift towards the virtual bodies, was only significantly modulated in the BS condition.

#### Questionnaire

**FIGURE 3:**
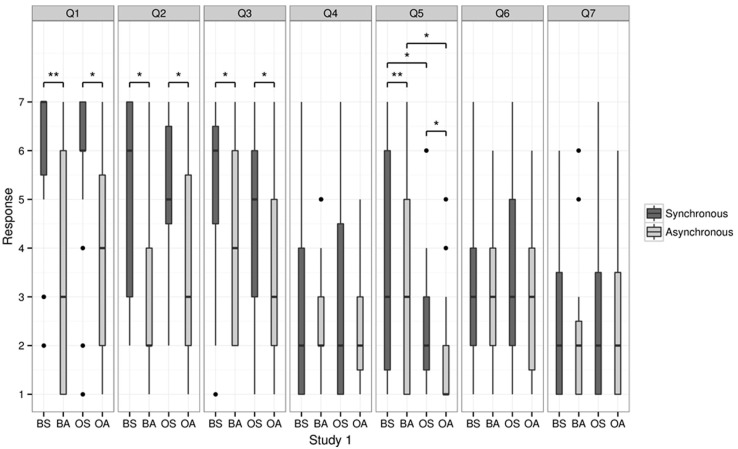
**Questionnaire results for Study 1.** This figure shows the median scores of the responses relating to touch (questions 1 and 2), self-identification (question 3), the sensation of having more than one body (question 5), as well as question 4, 6, and 7. Bold line indicates the median, upper and lower limit of the box plot indicate the upper and lower quartile (=75th and 25th percentile). Asterisks indicate a significant difference. BS = body synchronous, BA = body asynchronous, OS = object synchronous, OA = object asynchronous.

As predicted, self-identification with the virtual bodies (question 3; “I felt as if the virtual body/object was my body”) was highest in the BS condition and lowest in the OA condition (see **Figure 3**). Self-identification was rated significantly higher in the BS (median = 6) than in the BA (median = 4; *p* = 0.048). We also found that self-identification was higher in the OS (median = 5) than the OA condition (median = 3; *p* = 0.026). No significant difference was found between the BS and OS and BA and OA for question 3 (*p* > 0.14).

We found that the sensation of having more than one body (question 5) depended on synchrony and on whether a body or an object was shown: double body ownership was rated higher for the BS (median = 3) than for BA (median = 3, *p* = 0.01) and OS (median = 2) higher than OA (median = 1, *p* = 0.03). Moreover, the BS condition was rated higher than the OS (*p* = 0.02) and BA was rated higher than OA (*p* = 0.02, all *p* values corrected for multiple comparisons using the Holm–Bonferroni method).

Moreover, statistical analysis revealed significant differences between the four conditions for questions 1 and 2. Participants gave a significantly higher positive rating in the BS condition (BS, median = 7) compared to the BA (median = 3) condition for question 1 (“It seemed as if I was feeling the touch of the stick in the location where I saw the virtual body/object being touched”; *p* = 0.01) and for the OS (median = 6) compared to the OA (median = 4), respectively (*p* = 0.03). No significant difference was found between BS and OS and BA and OA, respectively (all *p* > 0.6). As for question 2 (“It seemed as though the touch I felt was caused by the stick touching the virtual body/object”) participants rated the BS condition significantly higher (median = 6) than the BA condition (median = 2; *p* = 0.035) and the OS significantly higher (median = 5) than OA (median = 3, *p* = 0.025).

In Question 8, 57% of the participants indicated that they identified with both virtual bodies equally (see **Figure 6**). The responses were considered spatially on an ordinal scale, such that left < both < right. There was a significant effect of *synchrony*, χ^2^(1) = 5.90, *p* = 0.015, such that participants’ responses were biased to the right in the synchronous conditions. The effect of *body* and the interaction effect were not significant (*p* > 0.77).

#### Self-location

**FIGURE 4:**
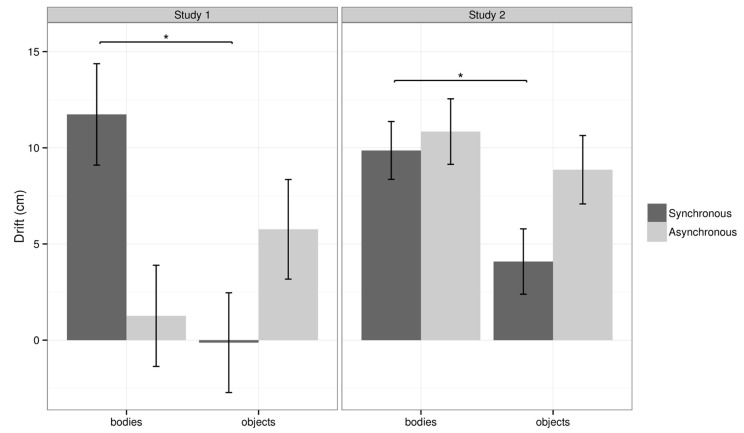
**Drift results for Study 1 and Study 2.** This figure shows the mean drift for BS, BA, OS, and OA for study 1 and study 2. Error bars represent one standard error of the mean. Asterisks indicate a significant difference based on pairwise comparisons. Note: the overall analysis showed a significant interaction between body and synchrony for study 1. The main effects and interaction effect were not significant for study 2, and the pairwise comparison was referred to in the discussion.

In the BS condition the participants showed a mean anterior–posterior drift in self-location of 11.7 cm towards the virtual bodies, whereas in the BA condition the mean drift was only 1.3 cm. In the OS conditions the participants showed a mean drift of -0.1 cm and in the OA a mean drift of 5.8 cm towards the objects (**Figure 4**).

The statistical analysis of the mean drift measure revealed a significant interaction between *body* and *synchrony*, χ^2^(1) = 5.23, *p* = 0.022, with no significant main effects (all *p* > 0.3). Separate analyses were conducted for each level of synchrony to explain the interaction effect. The analysis revealed a main effect of *body* was present in the synchronous conditions, χ^2^(1) = 5.60, *p* = 0.018 (more drift towards bodies compared to objects) but this was not significant in the asynchronous conditions. The left–right drift was not significantly modulated by experimental condition.

### STUDY 2

We found that self-identification depended on synchrony, irrespective of whether two virtual bodies or two virtual objects were presented. The sensation of having multiple bodies and self-location was not significantly modulated by the experimental conditions.

#### Questionnaire

**FIGURE 5:**
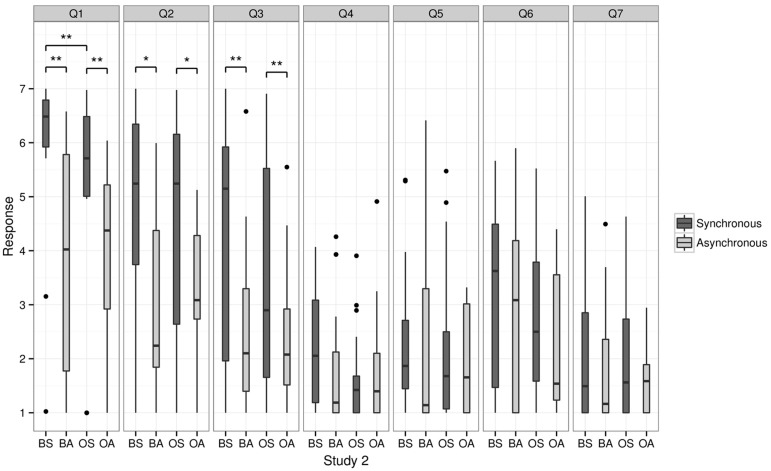
**Questionnaire results for Study 2.** This figure shows the median scores of responses relating to touch (questions 1 and 2), self-identification (question 3), the sensation of having more than one body (question 5), as well as question 4, 6, and 7. Bold line indicates the median, upper and lower limit of the box plot indicate the upper and lower quartile (=75th and 25th percentile). Asterisks indicate a significant difference. BS = body synchronous, BA = body asynchronous, OS = object synchronous, OA = object asynchronous.

A similar pattern concerning illusory self-identification (question 3) was found in study 2. Self-identification was rated significantly higher in the BS condition (median = 5.15) than in the BA (median = 2.10; *p* = 0.01, see **Figure 5**). Again, we found that self-identification was higher in the OS (median = 2.90) than the OA condition (median = 2.08; *p* = 0.009). No significant difference could be found between BS and OS (*p* = 0.06) and BA and OA (*p* = 0.69, all *p* values corrected for multiple comparisons using the Holm–Bonferroni method). In contrast to study 1, double body ownership (question 5) was not significantly modulated by the experimental conditions (*p* > 0.05).

The analysis of questionnaire results for study 2 further revealed significant differences between the four conditions for questions 1 and 2. Participants gave a significantly higher positive rating in the BS (median = 6.48) compared to the BA condition (median = 4.02) for question 1 (*p* = 0.004). Participants also rated the OS (median = 5.71) higher than the OA (median = 4.38; *p* = 0.009) for question 1. BS was rated significantly higher than OS (*p* = 0.01). No significant difference was found between BA and OA (*p* = 0.92).

For question 2 participants rated the BS (median = 5.24) significantly higher than the BA condition (median = 2.24; *p* = 0.02) and the OS (median = 5.24) significantly higher than OA condition (median = 3.09; *p* = 0.021). No significant difference was found between BS and OS (*p* = 0.47), and between BA and OA (*p* = 0.72).

**FIGURE 6:**
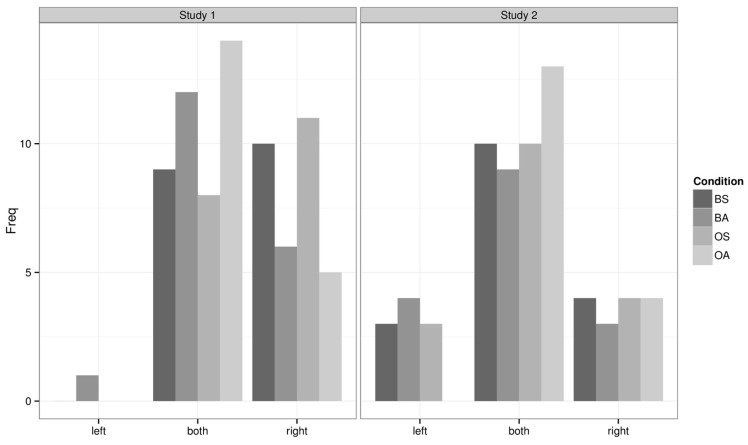
**Question 8 results for Study 1 and Study 2.** This figure shows the frequency counts for the response categories left, both, and right for question 8.

In Question 8, 54% of the participants indicated that they identified with both avatars/objects equally (see **Figure 6**). The analysis of the responses showed neither significant effects of *body* and *synchrony* nor a significant interaction effect (all *p* > 0.31). Importantly, of those who responded that they identified with both avatars equally, 75.6% reported that they identified with the avatars at the same time (Question 9). This was true irrespective of experimental condition, χ^2^(3) = 1.69, *p* = 0.64.

#### Self-location

In the BS condition the participants showed a mean anterior–posterior drift in self-location of 9.9 cm towards the virtual bodies, whereas in the BA condition the mean drift was 10.8 cm. In the OS conditions the participants showed a mean drift of 4.1 cm towards the objects and in the OA a mean drift of 8.9 cm towards the objects (see **Figure 4**).

The statistical analysis of the mean drift revealed no significant main effects of body [χ^2^(1) = 2.82, *p* = 0.093], synchrony [χ^2^(1) = 1.62, *p* = 0.20], nor an interaction [χ^2^(1) = 0.72, *p* = 0.40]. No significant left–right drift was found.

### BETWEEN-GROUPS COMPARISON

We conducted a between-groups statistical analysis to compare the results of the video-based VR technique (study 1) and the 3D computer generated VR (study 2). Our analysis had therefore three factors: *study*, *body* (bodies vs. objects) and *synchrony* (synchronous vs. asynchronous). *Study* was a between-subjects factor and *body* and *synchrony* were within-subjects.

#### Questionnaire

We conducted a principal components analysis (PCA) on the questionnaire results for questions 1–7 from both studies to identify the underlying components. We compare the results of question 8 separately, because it had responses on a different scale (responses were right, left or both).

For questions 1–7, data from both studies were taken into account (independent samples) and a preliminary analysis was carried out by running a PCA for each within-subject condition separately, to see if the structure was similar across conditions. Following this we report a complete PCA for the mean data from each participant across conditions to extract a single structure used to calculate factor scores. We then analyze the effect of *study*, *body*, and *synchrony*.

#### Preliminary Analysis

The PCAs were carried out using the psych package in R (R Core Team, 2013; Revelle, 2013). Bartlett’s test was significant in each within-subjects condition indicating that the correlation matrices differed from an identity matrix. The Kaiser–Meyer–Olkin (KMO) measure ranged from mediocre to middling (Kaiser, 1974) and were 0.66 (BS), 0.72 (BA), 0.68 (OS), and 0.66 (OA). The number of factors to extract was based on Kaiser’s criterion of choosing eigenvalues >1 (Kaiser, 1960), and confirmed using the scree plot. Rotation was oblique (oblimin) on theoretical grounds that the underlying factors may be related.

In general conditions yielded two factors, with questions relating to illusory *touch*, and questions related to illusory *drift*. The categories come from the questions themselves which primarily ask the participant about the touch of the stick (questions 1, 2, and 6), and drifting forwards or backwards (questions 4 and 7). The exceptions were questions 3 and 5 which asked whether the participants felt as if the two virtual bodies/objects were their body (question 3), or whether they felt they had more than one body (question 5). It was interesting therefore to see whether these key questions corresponded closely with touch or drift. Questions were considered primarily related to one factor when their standardized loading was > 0.3 for that factor and ≤ 0.3 for the other. This was typically the case for all questions except number 5 (more than one body).

In the BS condition, Questions 1, 2, and 6 were related to illusory *touch* (see **Table 1**) and loaded heavily on this factor, with standardized loadings 0.67, 0.86, 0.57 respectively. Question 3 (self-identification) was also in this category (standardized loading 0.84). Questions 7 and 4 were related to illusory *drift* (**Table 1**) and loaded on this factor at 0.86 and 0.85. The remaining question, number 5 (more than one body) loaded on both factors, at 0.31 and 0.65.

Results and factor loadings were similar in the OS condition, with questions dividing into illusory *touch* and *drift* and question 5 loading on both factors at 0.39 and 0.45. The BA condition was similar but with question 5 loading entirely on touch. OA on the other hand had the most differing structure. For OA three factors were extracted (due to eigenvalues >1 and the scree plot) and we identified these as *drift* (questions 7 and 4), *touch-identification* (primarily questions 2 and question 3), and *touch-location* (questions 1 and 6). Question 5 loaded on *touch-identification* at 0.72 and <0.3 on the others.

In summary the questionnaire structure was similar with two factors for conditions BS, BA, and OS. The self-identification question 3 related to *touch* and question 5 (more than one body) related to both factors in the synchronous conditions. Note that the differing structure arose in the objects/asynchronous *control* condition only.

#### Effect of Study, Body, and Synchrony on Questionnaire Responses

We carried out a PCA to look at the overall structure of the questionnaire, taking the mean results for each participant across conditions (an approach similar to Tajadura-Jiménez et al., 2012). This showed the underlying factors in the overall questionnaire responses.

Bartlett’s test was significant indicating the correlation matrix differed from an identity matrix, *p* = 2×10^-^^14^. The KMO was 0.70 (middling), and the determinant was fine, 0.03. Two factors were extracted, based on Kaiser’s criterion and the scree plot, and these were again illusory *touch* (questions 1, 2, 6) and illusory *drift* (questions 4 and 7). Interestingly, question 3 loaded highly on *touch* (0.86) but low on *drift* (0.03). Question 5 related to *touch* and *drift* fairly equally (0.49 and 0.46 respectively). The pattern and structure matrices are shown in **Table 2**.

**Table 2:** Principal component analysis.

Item	Description	Illusory touch	Illusory drift	Illusory touch structure	Illusory drift structure	*h*^2^
2	…The touch I felt was caused by the stick touching the two virtual bodies/objects.	**0.93**		**0.89**		0.81
3	I felt as if the two virtual bodies/objects were my body.	**0.86**		**0.87**	0.30	0.76
1	…Feeling the touch of the stick in the location where I saw the two virtual bodies/objects touched.	**0.69**		**0.72**	0.32	0.53
6	…The touch I was feeling came from somewhere between my own body and the two virtual bodies/objects.	**0.51**		**0.58**	0.36	0.37
5	It seemed as if I might have more than one body.	**0.49**	**0.46**	**0.63**	**0.61**	0.59
7	It appeared (visually) as if the two virtual bodies/objects were drifting backwards …		**0.98**		**0.93**	0.89
4	It felt as if my (real) body was drifting forwards (towards the two virtual bodies/objects).		**0.81**	**0.51**	**0.89**	0.85

*Items are sorted by their factor loadings from the third and fourth columns, which are taken from the pattern matrix. The fifth and sixth columns are the factor loadings from the structure matrix. Loadings <0.3 are not shown, and >0.4 are highlighted bold. h^2^ Represents the communalities.*

Questions 1, 3, and 6 had low loadings on the *drift* factor in the pattern matrix and increased (>0.3) loadings on the *drift* factor in the structure matrix. Question 4 showed increased loading on the *touch* factor in the structure matrix, but still loaded mostly on *drift*. The changes from the pattern to the structure matrix were a result of the relationship between the two factors. However, this relationship did not obscure the pattern of results: the question items divided into the two underlying factors of illusory touch and drift fairly consistently in the two matrices.

Factor scores for illusory touch and illusory drift were calculated for all participants in all conditions, from the structure matrix using the regression method (Field et al., 2012). The scores take into account the loading of each question onto each factor, and the relationship between pairs of questions.

A multivariate analysis of variance using Pillai’s trace, with experimental conditions *study*, *body*, and *synchrony*, showed a significant main effect of *study* on illusory *touch* and *drift*, *V* = 0.080, *F*(2,135) = 5.85, *p* = 0.0037, in that illusory *touch* and *drift* were both higher in study 1 (video-based) than study 2 (3D computer generated). There was also a main effect of *synchrony*, *V* = 0.16, *F*(2,135) = 12.96, *p* = 7.1 × 10^-^^6^, in that illusory *touch* and *drift* were higher in the synchronous condition.

Individual analyses were conducted separately on dependent variables *touch* and *drift* to understand whether the effects were predominantly taking place on one variable only. Two linear mixed-effects models were used (one for touch and the other for drift) with *study*, *body*, and *synchrony* as factors, and the latter two factors as nested random effects within subjects. The results for *touch* revealed a significant effect of synchrony, χ^2^(1) = 51.86, *p* < 0.0001, such that *touch* was higher in the synchronous conditions. An analysis on *drift* revealed a significant effect of body, χ^2^(1) = 4.78, *p* = 0.029, and synchrony, χ^2^(1) = 7.39, *p* = 0.0065, such that *drift* was higher in the body conditions compared to the objects and in the synchronous conditions compared to the asynchronous. All other effects were non-significant.

In summary, the effect of *study* was found in the multivariate analysis on the underlying questionnaire components *touch* and *drift*, indicating significantly stronger illusion responses from study 1 compared to study 2. But the study effect was only found when considering the two dependent variables together, suggesting there was an overall effect on the illusion that was not detected in a single component alone. The effect of *synchrony* was present in both dependent variables, *touch* and *drift*. The effect of *body* was found only in the follow-up analysis, on the illusory *drift* response. The bodily appearance is therefore relevant to the illusory drift, and this relates coherently to our findings from both studies using the behavioral drift measure of self-location (**Figure 4**).

#### Self-Location

A linear mixed-effects model comparing the effect of *study, body* (bodies vs. objects), and *synchrony* (synchronous vs. asynchronous) on anterior–posterior drift, with *body* and *synchrony* as random effects nested within subjects, showed a significant interaction between *body* and *synchrony*, χ^2^(1) = 5.76, *p* = 0.016. All other effects were non-significant. The analysis proceeded by looking at each level of synchrony separately, to understand the interaction.

At the synchronous level there was a main effect of *body*, χ^2^(1) = 9.40, *p* = 0.0022, such that we measured a self-location drift further forwards in the body condition (mean = 10.85 cm) compared to the object control (mean = 1.86 cm). At the asynchronous level the effect of body was non-significant. The effect of body on anterior–posterior drift revealed itself in the synchronous condition only.

There was a significant effect of *study* on left–right drift, χ^2^(1) = 6.39, *p* = 0.012, such that there was a bias to the right in study 2 (3D computer generated VR) mean = 6.06 cm to the right, compared to study 1 (video-based VR), mean = -4.11 cm.

#### Identification with Left, Right, or Both Bodies

Question 8 on the questionnaire was considered spatially as an ordinal response, in that left < both < right. We analyzed the responses as before using proportional-odds logistic regression (McCullagh and Nelder, 1989). The experimental factors were *study*, *body*, and *synchrony*. The data are plotted in **Figure 6**. We can see the data in study 2 appear more centralized and so we adopt this as our base study. This does not change the results of the analysis but makes the coefficients straightforward to interpret, such that a positive coefficient indicates a rightward bias compared to the more centralized distribution. The analysis resulted in a significant main effect of *study*, χ^2^(1) = 13.05, *p* = 0.0003, with a standardized coefficient *Z* = 2.26. This indicated that there were significantly more rightward responses in study 1 (video-based VR) compared to study 2 (3D computer generated VR). The effects of experimental factors *body* and *synchrony* were not significant.

The results suggest there is an underlying rightward bias present in both studies that has revealed itself statistically in the between-groups comparisons in study 1 in the questionnaire, and in study 2 in the self-location drift.

## DISCUSSION

We report that self-identification with two virtual bodies was stronger during synchronous stroking as compared to asynchronous stroking. This was accompanied by the sensation of having more than one body and a change in self-location towards the virtual bodies that was body-specific and depended on the synchrony of stroking in study 1. We discuss (1) the implication of our findings for the study of bodily self-consciousness, (2) possible explanations for the differing results in study 1 and 2, and (3) the implications for the use of two widely used VR techniques to investigate visual capture illusions.

### EXPERIENCE OF HAVING TWO BODIES

Using a modified version of the full body illusion paradigm in which we presented two virtual bodies, we demonstrate that it is possible to self-identify simultaneously with two virtual bodies. This is in contrast to the classical setup, where participants are exposed to a single second body (the virtual body) in addition to their own physical body, but do not report having more than one body (Lenggenhager et al., 2007). The present data are a first step towards the investigation of double body ownership, e.g., to feel being touched on two visually presented bodies and to identify with the two bodies simultaneously. Interestingly, in study 1, this was further associated with the subjective feeling of having multiple bodies.

Similarly, the rubber hand illusion has been recently extended to more than one fake hand: whereas in the classical rubber hand illusion setup it has been argued that the feeling of illusory ownership is limited to the fake (seen) hand (Botvinick and Cohen, 1998), it has been shown that the use of two fake hands results in the sensation of having an additional hand (Newport et al., 2010), as can be observed in patients suffering from a supernumerary phantom limb of neurological origin (Khateb et al., 2009). Furthermore, Guterstam et al. (2011) showed that healthy participants experience a second additional right hand (“supernumerary limb illusion”) if the real hand was visible during the rubber hand illusion. Questionnaire and skin conductance response data (SCR) provided further evidence that ownership was equal for the real and the fake hand, in contrast to the significant disownership for the real hand which accompanies the traditional rubber hand illusion (Moseley et al., 2008). The authors suggest that this is achieved through multisensory integration processes in premotor and parietal cortices representing two equally probable locations for the seen somatosensory stimulation and therefore identifying with the two hands at the same time. One might argue that our findings could be interpreted in a similar way. However, there are some important differences between the present setup and the one used by Guterstam and colleagues. First, while being exposed to two virtual bodies through the HMD, the physical body was not directly visually perceived during our experiment. We argue that the visual presentation of two virtual bodies is necessary to induce “double” body ownership, as earlier work using the traditional full body illusion (with one virtual body) did not induce the sensation of having two bodies (Lenggenhager et al., 2007). Therefore, our study is closely related with the experimental setup of Ehrsson (2009) investigating the “supernumerary limb illusion,” where also two fake hands are presented while the physical hand is covered. We speculate that the experience of self-identification with both virtual bodies is due to the extension of the large and often bilateral receptive fields in bimodal visuo-tactile neurons in the parietal cortex to include both virtual bodies (i.e., Duhamel et al., 1998; Blanke, 2012). Such bimodal visuo-tactile neurons have been shown to integrate visual stimuli in peripersonal space and tactile stimuli applied to the upper body and face. Secondly, there is an important difference between the sensation of a supernumerary body part and the sensation of double body ownership: while one can experience having an additional limb, the singularity of self-awareness might still be conserved, e.g., there remains a singular self that seems to have a total of three limbs during the illusory state.

The identification with more than one body and the sensation of having more than one body, as observed in study 1 and not confirmed in study 2, should, however, be regarded as a preliminary experimental step towards understanding the mechanisms involved in the experience of the singular self and its loss. We note that double body ownership has been reported clinically in cases of heautoscopy for several decades (Brugger, 2002; Blanke et al., 2004; Heydrich and Blanke, 2013). The present experiment failed to evoke behavioral measures of such strong duplications of the self as behavioral drift in self-location towards the virtual bodies (in study 1) was similar to the effect of viewing a single body (Lenggenhager et al., 2007; Ionta et al., 2011). More work, likely using additional interoceptive manipulations (i.e., Aspell et al., 2013; Suzuki et al., 2013), may be necessary to achieve stronger distortions of the self, associated with double body ownership as in heautoscopy (Brugger, 2002; Heydrich and Blanke, 2013). Although we argue that the drift measure reflects the position in space (self-location) where the subjects felt that they were standing during the illusion and note that it has been replicated across several studies (Lenggenhager et al., 2007; Aspell et al., 2009) and using different techniques (e.g., mental imagery, Ionta et al., 2011), the drift measure by itself is not an objective measure for double body ownership. However, future research will have to address the question of how viewing two bodies affects changes in self-location, also in the right–left axis, more systematically, e.g., by stroking one body synchronously while stroking the other body asynchronously, or by using eye-tracking to observe whether participants’ attention could be biased towards one body.

There are also several differences and limitations in our findings if compared with those obtained in previous experiments using the full body illusion. First, in contrast to the findings of Lenggenhager et al. (2007), modulation of illusory self-identification by synchronous visuo-tactile stimulation in the present study was not limited to the body condition; thus participants reported that they also self-identified with the two objects (question 3). This is in line with findings from Armel and Ramachandran (2003), who showed that participants showed increased SCR and ownership ratings when the “RHI” was performed with a table. This occurred when the table was stimulated synchronously with the subject’s hand, and in absence of a fake hand. We note that, since participants in our studies were required to fixate on a cross between the two bodies (or two objects), this might have also reduced the body specificity of the illusions (e.g., self-identification) as reported previously (Lenggenhager et al., 2007). In line with this argument is the observation that in a recent experiment by Aspell et al. (2009) using the full body illusion and the CCE, the specificity of the full body illusion as measured by questionnaires was also not limited to the virtual body, e.g., synchronous visuo-tactile stimulation also modulated self-identification with an object. It could be argued that the CCE, an attention demanding discrimination task (i.e., a dual task next to the full body illusion), may reduce the body-specificity often observed in the absence of such dual tasks. As neither Ehrsson (2009), Guterstam et al. (2011), nor Newport et al. (2010) reported an object control condition or another attention demanding task during their experiments with an extra hand, it is not known whether self-identification for the two objects in the present experiment was due to experimental differences or due to the fact that in our studies two bodies/objects were presented simultaneously.

The overall findings from study 1 were replicated to some extent in our second study, using 3D computer generated graphics. Self-identification with the two virtual bodies was positively modulated by visuo-tactile synchrony, irrespective of whether two bodies or two objects were presented (question 3). Likewise, illusory touch and illusory causation (questions 1 and 2) were induced by visuo-tactile synchrony. Importantly, we found that participants identify with the two bodies at the same time, e.g., we specifically asked participants in study 2 if they identified with the two virtual bodies simultaneously or in an alternating fashion, and 76% of responses indicated that this double self-identification was happening simultaneously. However, we found that in the 3D computer generated setup participants showed a forward drift in self-location (towards both the avatars/objects) regardless of whether visuo-tactile stimulus was synchronous or not. Also, no sensation of having multiple bodies was reported in study 2.

Statistically, there were significantly stronger results on the underlying components of the questionnaire, namely illusory touch and illusory drift, in study 1 compared to study 2. The effect of *body* was in particular important to illusory drift. This was consistent with the findings from the overall behavioral drift: an effect of *body* was found on the behavioral drift in the synchronous conditions. The between-groups comparison also revealed a rightward bias present in both studies, that was observed in the questionnaire results (study 1) and in the behavioral drift measure (study 2), so in this sense the overall left–right drift results were similar in both studies.

The partial discrepancy between the results of study 1 and study 2 leads to the second part of the discussion, namely the comparison between the two VR techniques, which might explain the diverging results of study 1 and 2.

### COMPARISON BETWEEN VIRTUAL REALITY TECHNIQUES

The two studies used video and computer generated HMD virtual environments respectively, with the second study using motion tracking and 3D computer generated graphics to render the virtual bodies and stick.

In the following we would like to discuss several issues that may be of relevance when using VR techniques to study bodily self-consciousness, namely distance estimation, visual fidelity, latency, visual realism, and the self-location measure.

Distance estimation has been shown to be veridical with real-world expectations in carefully calibrated video HMD setups, but underestimated in computer generated HMDs (Swan Ii et al., 2006). An underestimation could have caused a change in our self-location measure. We note, however, that in this case self-location results should have been influenced globally across all conditions. In addition, both Mohler et al. (2010) and Ries et al. (2009) have demonstrated that seeing a virtual avatar in HMDs for a short time leads to no underestimation of distances in computer generated HMDs. For these reasons we do not think that distance underestimation played a role in our experimental results, or the differences between the results in study 1 and 2

Further differences between study 1 and study 2 were visual fidelity, visual realism and latency. Here, visual fidelity refers to whether the virtual body resembles the body of the participant. The virtual bodies in study 1 were video images of the participants, and in contrast study 2 used generic 3D avatars.

Although it seems that in study 2 no high visual fidelity was needed to identify with a virtual body or to some extent even with an object (question 3), it has been shown that top–down processes such as visual identity might influence the rubber hand illusion (Tsakiris and Haggard, 2005) and the full body illusion or related paradigms. This could explain the fact that we did not find a change in self-location and no illusory experience of ownership of more than one body in study 2.

Numerous object controls in previous studies have emphasized the relationship between appearance and illusory response, e.g., Lenggenhager et al. (2007) did not detect drift with an object control, and Petkova and Ehrrson (2008) found that greater visual fidelity mattered for their measure of skin conductivity in response to a visual threat, when they compared a human mannequin to an object control. Taking our results in the context of related work suggests that fidelity is more important for certain measures. From our results, those measures are self-location drift (consistent with Lenggenhager et al., 2007) and the subjective sensation of having more than one body, where the effects were only detected using the high fidelity visual bodies from study 1.

Object control conditions reinforce the principle that the objects to be embodied must look human-like. However, our results raise the question of whether there is a *fidelity scale*, in which higher fidelity objects produce stronger illusions of identification and ownership. Tsakiris et al. investigated this for the RHI with a range of hand-like objects from low (block) to medium (hand shape) to high fidelity (rubber hand). In general the principle was shown to hold, where increasing fidelity appeared to increase the illusion for a variety of measures. Interestingly, consistent with our findings, the effect of drift was particularly sensitive, and only the highest fidelity synchronous condition showed significant drift from the baseline measure (Tsakiris et al., 2010). Similarly, while participants reported ownership over two hand images in a study by Newport et al. (2010) this did not transfer to a motor response task. Thus, when visual fidelity is higher (study 1), we see a positive drift towards the virtual bodies and a significant effect of double body ownership in the body-synchronous condition (question 5), compared to the body-asynchronous control. However, it remains speculative why self-location and double body ownership (question 5) are more sensitive to visual fidelity as compared to illusory touch (question 1 and 2) and self-identification (question 3). For self-location, this may be because the effect can dissociate from self-identification, as suggested by Rohde et al. (2011) in the RHI. Their results for the RHI show that drift can be driven with vision only, results which in our context would predict the importance of visual fidelity to the drift measure. They found that drift can also occur in the asynchronous condition even when self-identification is low, i.e., the results we obtained in the body condition in study 2, which we discuss further below with regards to latency. Regarding double body ownership (question 5), further studies are needed to investigate the relationship with visual fidelity by directly comparing different visual renderings with the same methodology.

Another aspect of the computer generated setup is visual realism. Although full-body and body part illusions have been performed in other VR setups without high visual fidelity, there were still many differences between our study and previous implementations. For example, the plain background in study 2 is in stark contrast to the visual realism in a study from Slater et al. (2010), who created visual capture with a virtual environment consisting of a detailed scene, complete with fireplace, television, sound, virtual mirror, and interaction, i.e., when participants moved their head they could look around, which was not the case in the studies presented here. Any one of the additional details could be used by computer generated VR to increase visual capture, presence, and other factors. Thus, it may be necessary to add more details to the computer-generated environment to elevate the illusion quality and strength to those obtained with video (or to create an equal illusion of presence to that achieved by video). However, understanding the importance of each component in the VR setup, e.g., sound, 3D model, interaction, would require future, more systematic, work. The work would build on the findings here, suggesting that the similar experimental conditions using two different VR technologies show stronger results in video-based VR.

The final major difference between our two studies was latency. Although the delay between the stick physically touching the body and the visual stick touching the virtual body was only approximately 25 ms longer in study 2 than study 1, this increased latency could have reduced the difference between the synchronous and asynchronous conditions. Importantly, according to a recent review on intersensory synchrony, delays of >20 ms do become noticeable (Vroomen and Keetels, 2010). Rohde et al. (2011) showed a positive drift can be also found during the asynchronous condition in the RHI, i.e., changes in perceived hand position still occurred with asynchronous stroking. If we analyze, for example, the self-location measure in study 2 without the asynchronous conditions and compare the BS to the OS condition, then we do in fact find a significant drift towards the virtual bodies in the body condition as compared to the object condition [*t*(16) = 2.37, *p* = 0.03]. This would support the hypothesis that a synchrony-dependant change in self-location was not found in study 2 due to a weaker contrast between synchronous and asynchronous conditions and that mostly a difference between the body and the object condition was observed. A solution to this weaker contrast in study 2 would be to create a more delayed asynchronous condition, or alternatively to use a more sensitive measure of self-location. Already Lenggenhager et al. (2007) have speculated on the importance of their asynchronous control condition, and their first experiment did not use a delay in the asynchronous condition, but a pre-recorded video, which they note would be perceived as even more asynchronous and less predictable, thus creating a stronger contrast between synchronous and asynchronous experimental conditions. Similarly, Sanchez-Vives et al. (2010) used pre-recorded animation in their asynchronous condition, in a computer graphics version of the RHI. In addition, more robust behavioral measure of self-location might be needed, such as the CCE (Aspell et al., 2009), distance estimation measures (Ionta et al., 2011; Pfeiffer et al., 2013), or motor imagery (Ionta et al., 2012) as these are based on repeated reaction time measures (Aspell et al., 2009).

Additionally, we suggest that the illusion may be harder to detect with two bodies. Related work with lower fidelity setups or similar VR setups with equivalent latency used one body or part of the body. A combination of higher fidelity, higher realism, and lower latency may be demanded in the case of study 2 to detect the effect of self-location with more than one virtual body.

Finally, we note that a within-subject design would have given more statistical power in a comparison between the two techniques used. Unfortunately this was not possible, given that the technique for study 1 was only available in Lausanne and the technique for study 2 only in Tübingen. Although we tried to replicate the setup as close as possible, the above outlined comparison of the two techniques is therefore based on a between-subject design and some statistically significant differences may have been missed.

## CONCLUSION

We conclude from these experiments that participants can identify with two virtual bodies (study 1 and 2), if synchronous visuo-tactile stimulation is applied. Moreover, they can experience the sensation of having more than one body (as is evidenced in Study 1) and identify with the two bodies at the same time (as is reported in Study 2). In addition, changes in self-identification in study 1 are to some extent supported by a body-selective and stroking dependant drift in self-location towards the virtual bodies, although no objective measure of double body ownership was obtained. Comparing the results across studies showed significantly stronger responses in study 1 on the two underlying components of the questionnaire, illusory touch and illusory drift, indicating a stronger subjective illusion in the video-based VR. These data show a first step towards studying double body ownership experimentally, using paradigms from the field of bodily self-consciousness.

Finally, we suggest that the differences we found between video VR and 3D computer generated VR should be considered in further investigations. Computer generated techniques are useful in that many potentially interesting factors, i.e., identity, visual motor synchrony, and the number of virtual bodies are easy to implement and manipulate in a controlled manner. However, the visual realism of the computer generated environment needs to be rich enough to create a sense of presence in the virtual space. Specifically, our results suggest that visual fidelity is important for experiencing more than one body. Additionally, we propose that a strong contrast between the timing in the experimental and control conditions, as well as a more sensitive measure of self-location might be needed in order to use computer generated VR in place of video HMD technology to investigate visual capture and to study illusory body ownership of more than one body.

## Conflict of Interest Statement

The authors declare that the research was conducted in the absence of any commercial or financial relationships that could be construed as a potential conflict of interest.

## AUTHOR CONTRIBUTIONS

Lukas Heydrich, Trevor J. Dodds, and Jane E. Aspell have conducted the experiment and have analyzed the data, Lukas Heydrich, Trevor J. Dodds, Betty J. Mohler, Jane E. Aspell, Heinrich H. Bülthoff, and Olaf Blanke have designed the experiment, Lukas Heydrich, Trevor J. Dodds, Betty J. Mohler, Jane E. Aspell, and Olaf Blanke have written the manuscript.
